# Intermediate Bandgap (IB) Cu_3_VS_x_Se_4−x_ Nanocrystals as a New Class of Light Absorbing Semiconductors

**DOI:** 10.3390/nano16020082

**Published:** 2026-01-07

**Authors:** Jose J. Sanchez Rodriguez, Soubantika Palchoudhury, Jingsong Huang, Daniel Speed, Elizaveta Tiukalova, Godwin Mante, Jordan Hachtel, Arunava Gupta

**Affiliations:** 1 Department of Chemistry and Biochemistry, The University of Alabama, Tuscaloosa, AL 35401, USA; jjsanchezrodriguez@crimson.ua.edu (J.J.S.R.);; 2 Chemical and Materials Engineering, University of Dayton, Dayton, OH 45469, USA; manteg1@udayton.edu; 3 Center for Nanophase Materials Sciences, Oak Ridge National Laboratory, Oak Ridge, TN 37830, USA; huangj3@ornl.gov (J.H.); tiukalovae@ornl.gov (E.T.);; 4 Department of Chemical and Nano Engineering, University of California San Diego, La Jolla, CA 92093, USA

**Keywords:** intermediate band gap nanocrystals, Cu_3_VS_x_Se_4−x_, DFT, thin film solar cells

## Abstract

A new family of highly uniform, cubic-shaped Cu_3_VS_x_Se_4−x_ (CVSSe; 0 ≤ x ≤ 4) nanocrystals based on earth-abundant materials with intermediate bandgaps (IB) in the visible range is reported, synthesized via a hot-injection method. The IB transitions and optical band gap of the novel CVSSe nanocrystals are investigated using ultraviolet-visible spectroscopy, revealing tunable band gaps that span the visible and near-infrared regimes. The composition-dependent relationships among the crystal phase, optical band gap, and photoluminescence properties of the novel IB semiconductors with progressive substitution of Se by S are examined in detail. High-resolution transmission electron microscopy and scanning electron microscopy characterization confirm the high crystallinity and uniform size (~19.7 nm × 17.2 nm for Cu_3_VS_4_) of the cubic-shaped nanocrystals. Density functional theory (DFT) calculations based on virtual crystal approximation support the experimental findings, showing good agreement in lattice parameters and band gaps across the CVSSe series and lending confidence that the targeted phases and compositions have been successfully realized. A current conversion efficiency, i.e., incident photon-to-current efficiency, of 14.7% was achieved with the p-type IB semiconductor Cu_3_VS_4_. These novel p-type IB semiconductor nanocrystals hold promise for enabling thin film solar cells with efficiencies beyond the Shockley–Queisser limit by allowing sub-band-gap photon absorption through intermediate-band transitions, in addition to the conventional direct-band-gap transition.

## 1. Introduction

With the rising global energy demand, there is a continuous effort to achieve more sustainable avenues for energy conversion. Solar photovoltaics (PV) is a promising option among them, offering a clean and efficient way to harness energy from sunlight [1,2,3]. Thin film solar cells consisting of absorber layers processed from nanocrystal solutions are attractive among PV technologies for their tunable absorption in the visible spectrum and economic viability [4,5]. To date, copper-based ternary chalcogenide semiconductors, specifically Cu(In_x_Ga_1−x_)S_2_ (CIGS), have achieved the highest energy conversion efficiency in thin film solar cells [6]. However, indium is not widely available on earth’s crust and efforts to replace it led to alternate absorber layer materials such as Cu_2_ZnSnS_4_ (CZTS), but at the cost of reduced overall efficiency of the solar cells [7]. We have previously reported new wurtzite-phase materials, Cu_2_Zn(Al/Ga/In)S_4−x_ (CZAS) and CuZn_2_AS_4_, which partially replaced the In with Zn [8]. However, Cu_3_VS_4_, a p-type semiconductor of the sulvanite family, offer a more attractive alternative material for solar cells as they consist of entirely earth-abundant elements and exhibit intermediate band gap in the visible range (~1.3 eV) [9,10]. The sulvanites have low hole effective mass, which is beneficial for absorber layers. Moreover, Cu_3_VS_4_ is predicted to have intermediate bands (IB), making it a unique class of material for solar cells where the mid-gap states can potentially facilitate enhanced energy conversion efficiency beyond the traditional Shockley-Queisser limit (63.1%) [11,12]. These IB materials are distinct from the conventional direct-band-gap absorber materials, such as CZTS and Sb_2_(S,Se)_3_, in that they can absorb photons of energy equal to the mid-gap energy states, which facilitates transitions of charge carriers within the sub-band-gap states in addition to the transitions from valence to conduction band states allowed in the direct-band-gap materials [13,14]. Consequently, the photogenerated current in the IB solar cells is theoretically higher than that of the direct-band-gap solar cells. Therefore, in addition to the contribution in absorption from high energy photons of energy closer to the direct band gap, sub-band-gap energy photons can be absorbed in these IB semiconductors to promote transition of electrons from the valence band (VB) to the conduction band (CB) through the IB.

The bulk form of Cu_3_VS_4_ has been previously synthesized via solid-state approaches [15]. Additionally, pulsed laser deposition has been used for preparing thin films of Cu_3_VS_4_ [16]. However, nanoscale synthesis offers a higher degree of control over crystallinity, composition, crystal phase, shape, and size, enabling the tuning of electronic structures with greater precision. To this end, Chen et al., have used a solvothermal route for the synthesis of sulvanite nanocrystals (NCs), followed by a sulfide-based ligand exchange method, although IBs of the product have not been reported [17]. Increased tunability in edge length of cubic-shaped Cu_3_VS_4_ NCs within the range of 9–18 nm and subsequent control over intermediate bands have been achieved by Mantella et al., via a hot-injection approach [18]. Furthermore, the hot-injection route has also been successfully employed to synthesize cubic Cu_3_VSe_4_ p-type semiconductor NCs, which exhibit a photocurrent of ~4 μA/cm^2^ in an electrochemical measurement setup [19]. These reports highlight the growing interest in sulvanite materials for harnessing solar energy. Meanwhile, S/Se alloying has emerged as another powerful strategy for tuning structural and optoelectronic properties, providing an additional degree of freedom for band gap engineering and intermediate band optimization. Such alloying enables gradual modulation of lattice constants, band dispersion, and defect states, as demonstrated in recent studies [20,21]. Nevertheless, synthesizing sulvanite NCs with high uniformity of size, shape, and crystallinity has proven to be a significant challenge to date.

In this study, we report the colloidal synthesis of a novel class of highly uniform, cube-shaped Cu_3_VS_x_Se_4−x_ NCs and investigate their structure-property relationships. The key trends in optical band gap, crystal structure, and visible range photoluminescence (PL) are systematically investigated in the novel CVSSe NCs as a function of Se chalcogen substitution, using a combination of UV-vis spectroscopy, PL spectroscopy, and X-ray diffraction (XRD) techniques, to elucidate the structure-property relationships of these intermediate bandgap (IB) semiconductor NCs. The phase purity of Cu_3_VS_x_Se_4−x_ NCs is thoroughly assessed by means of XRD, high-resolution transmission electron microscopy (HRTEM), and atomic resolution scanning transmission electron microscopy (STEM). To complement the experimental characterization, density functional theory (DFT) calculations using the virtual crystal approximation were performed, showing good agreement with measured lattice parameters and band gaps across the CVSSe series. These theoretical results lend additional support to the phase purity and compositional control achieved in the synthesized nanocrystals. The unique optical characteristics of the NCs, including VB to IB transitions, are investigated experimentally via ultraviolet-visible spectroscopy (UV-vis). Additionally, we examine the significant photoresponse characteristics of Cu_3_VS_4_ NCs, which are of particular interest for photovoltaic applications.

## 2. Experimental

### 2.1. Materials

Copper iodide (CuI, ≥98.0%), copper acetylacetonate (Cu(acac)_2_, 98.0%), vanadium acetylacetonate (V(acac)_3_, 98%), analytical grade hexane, and ethanol were obtained from Sigma-Aldrich (MilliporeSigma, Burlington, MA, USA). 1-octadecene (ODE, technical grade, 90%) and 1-dodecanethiol (1-DDT, 98%) were obtained from Alfa Aesar (Alfa Aesar, Ward Hill, MA, USA). Oleylamine (OLA) of distilled quality (min 99%) was obtained from Silver Fern (Silver Fern Chemical Inc., Seattle, WA, USA). All chemicals were used as received without any further purification.

### 2.2. Synthesis of Cu_3_VS_4_ NCs

Cu_3_VS_4_ NCs were synthesized by modifying the protocol previously reported by Mantella et al. [18,22,23]. In a typical synthesis, 0.25 mmol of CuI and 0.33 mmol of V(acac)_3_ were dissolved in ODE (7 mL) inside a nitrogen-filled glovebox and added to a four-neck flask connected to a Schlenk line. The mixture was stirred and heated to 280 °C. In a separate vessel, 10 mmol of 1-DDT and 1 mL of OLA were degassed under a cycle of vacuum and nitrogen at room temperature for 10 min. The thiol-containing solution was injected into the metal precursor solution at 280 °C. After injection, the reaction temperature was allowed to recover and held at 280 °C for 30 min. The resulting dark solution was allowed to cool down naturally and divided into two flasks with 20 mL of hexane/ethanol mixture (1:1 *v*/*v*) each. The final product (~10 mg) was collected by centrifugation at 5000 rpm for 15 min. We also synthesized Cu_3_VS_4_ NCs using Cu(acac)_2_ precursor instead of CuI, keeping all other parameters and the synthesis process the same.

### 2.3. Synthesis of Cu_3_VS_x_Se_4−x_ NCs

In a typical synthesis of Cu_3_VS_x_Se_4−x_ NCs (i.e., Cu_3_VS_3_Se, Cu_3_VS_2_Se_2_, Cu_3_VSSe_3_, and Cu_3_VSe_4_) containing both S and Se chalcogen anions, 0.75 mmol of the Cu precursor, CuI and 0.75 mmol of the V precursor, V(acac)_3_, and 7 mL of the solvent, ODE were stirred in a 3-neck flask for 30 min under N_2_ purge in a Schlenk line system and then heated to 280 °C. In another flask, the chalcogen precursors, i.e., 1-DDT (10 mmol) and stoichiometric quantity of diphenyl diselenide were stirred for 30 min at room temperature in the solvent and ligand, OLA (1 mL) under an inert atmosphere. The chalcogen solution was then injected into the reaction mixture containing the metal precursors at 280 °C. The reaction temperature was allowed to recover after the injection prior to heating the reaction at 280 °C for 30 min to form the CVSSe NCs. The NC solution was then allowed to cool and was purified via centrifugation, following a procedure similar to the one used for the Cu_3_VS_4_ NCs. The yield from each synthesis reaction batch is ~10 mg of CVSSe NCs. CVSSe NCs were also synthesized using Cu(acac)_2_ as the copper precursor instead of CuI, keeping all other conditions the same.

### 2.4. Characterization

The crystallographic analysis of the CVSSe NCs was performed by X-ray diffraction using a BRUKER D2 diffractometer (Bruker Co., Billerica, MA, USA) with a Cu Kα (1.5406 Å) radiation source. The absorbance spectra were measured using a Shimadzu UV-3600i Plus UV-vis-NIR spectrophotometer (Shimadzu Co., Kyoto, Japan), and the optical transitions were assessed from the Tauc plot analysis. Scanning electron microscopy (SEM) analysis was carried out using an Apreo FE-SEM (Thermo Fisher Scientific Inc., Waltham, MA, USA) to probe the surface morphology and thickness of the fabricated thin films. Well-dispersed solutions of the CVSSe NCs were prepared in hexane at concentrations ~ 1.5 gL^−1^ via 30 min sonication at room temperature (Branson 1800, Fisher, USA) to form the samples for PL studies. The PL analysis over the visible range was conducted on a Fluoromax spectrofluorometer (Horiba Scientific, USA). The emission spectra of the NCs were measured in the visible 360–1100 nm range at increments of 0.5 nm for a 350 nm excitation source. Transmission electron microscopy (TEM) and high-resolution TEM (HRTEM) analyses were performed using an FEI Tecnai 20 (Thermo Fisher Scientific Inc., Waltham, MA, USA) with a 200 kV operating beam. Atomic resolution Scanning transmission electron microscopy (STEM) analysis was performed using a Nion Hermes (Nion Company, Kirkland, WA, USA) monochromated aberration-corrected (MAC) STEM. The samples were dispersed in hexane and dropped onto a carbon-coated gold grid for TEM and STEM analysis. The samples were baked at 160 °C under vacuum for 24 h before analysis. The elemental composition was determined using energy-dispersive X-ray analysis (EDX). The photoresponse of the fabricated thin films was investigated by performing current-voltage (I–V) characteristics measurements in dark and under one sun (100 mW cm^−2^, AM 1.5 G) illumination conditions using a Princeton potentiostat (AMETEK Inc., Berwyn, PA, USA) in the applied potential window of −500 mV and +500 mV.

### 2.5. Computational Studies

DFT calculations were performed using the Vienna ab initio simulation package [24,25]. Kohn-Sham equations were solved by using a projected-augmented wave (PAW) method. Structural relaxations for both lattice vectors and atomic positions of the cubic structure retrieved from the Materials Project database were performed employing the Strongly Constrained and Appropriately Normed (SCAN) meta-Generalized Gradient Approximation (GGA) functional, in conjunction with the virtual crystal approximation (VCA) to address the S/Se alloying in Cu_3_VS_x_Se_4−x_ [26,27,28]. The relaxed structures were then used for electronic structure calculations employing the Heyd–Scuseria–Ernzerhof (HSE06) hybrid functional [29]. The kinetic energy cutoff for plane waves was set to 550 eV and the Brillouin-zone integrations were performed on Γ-centered 12 × 12 × 12 k-point grid. The convergence criteria for the structural relaxations and electronic self-consistency were, respectively, set to a Hellmann-Feynman force of 10^−2^ eV/Å and energy difference of 10^−5^ eV, and the “accurate” precision setting was adopted to avoid wrap around errors. Band structures were sampled along the path specified in [28,30].

## 3. Results and Discussion

### 3.1. XRD Analysis of Cu_3_VS_x_Se_4−x_ NCs

**Figure 1:**
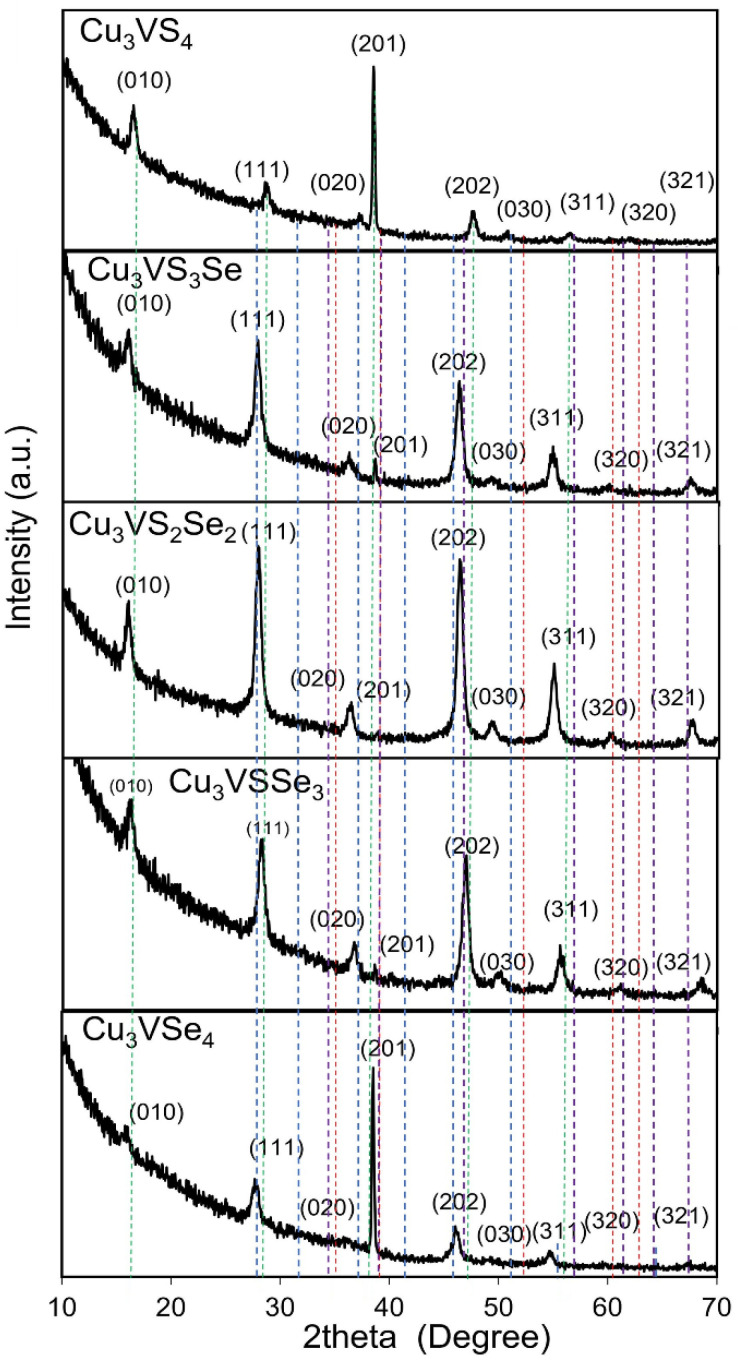
XRD plots for various Cu3VSxSe4−x NC compositions exhibiting a cubic P$\overset{-}{4}$3m phase. The background signal arising from the glass slide used to mount the NC samples has not been subtracted. The peak positions for CuI are shown as blue dotted line on the plot for comparison. Peak positions for zinc blende phase of CuS are shown as red dotted line on the plot for comparison. Peak positions for wurtzite phase of CuS are shown as violet dotted line on the plot for comparison. The green dotted lines show the (010), (111), (201), (202), and (311) crystal planes of Cu3VS4 NCs and demonstrate the peak shift with substitution of Se for the CVSSe NC compositions.

Figure 1 shows the XRD characterization of Cu_3_VS_x_Se_4−x_ NCs [31]. The XRD structure of the Cu_3_VS_4_ NCs reveals a cubic crystal phase of space group, P$\bar{4}$3m (No. 215) and a lattice parameter (*a*) of 5.396 Å, which is in close agreement with reported literature [32,33]. The major diffraction peaks at 2θ angles of 14°, 29°, 34°, 38°, 48°, 51°, and 58° are indexed to (010), (111), (020), (201), (202), (030), and (311) crystal planes of the pure cubic phase. Furthermore, the average particle size of the NCs is estimated using the Scherrer formula, which relates the size of the nanocrystallites in a solid to the broadening of a peak in the diffraction pattern [34,35]:

$$D=\frac{0.94\lambda }{\beta cos\theta }$$

where λ is the wavelength of the X-ray radiation source Cu-Kα (1.5418 A), *β* is the full width at half maximum (FWHM) corresponding to all the peaks in the diffraction pattern, *θ* is the diffraction angle for lattice planes, and *D* is the particle diameter. The crystallite size is determined by averaging the values calculated from all the diffraction peaks present in the XRD pattern, giving an estimated particle size of 25.3 ± 8.3 nm for the Cu_3_VS_4_ NCs.

The various intermediate band gap semiconductor compositions of Cu_3_VS_x_Se_4−x_ NCs also exhibit a cubic P$\bar{4}$3m crystal phase, as evident from the XRD characterization (Figure 1). In addition, the XRD peaks corresponding to the different crystal planes of the pure cubic phase show a shift to lower 2θ angles with increase in Se substitution in the CVSSe compositions. This trend is expected due to the lattice expansion caused by the substitution of larger Se atoms for smaller S atoms in the CVSSe crystal lattice. However, in addition to compositional effects, the size of the NCs also influences the XRD patterns, giving rise to size-induced peak broadening and subtle shifts in peak positions. For example, the smaller size of the Cu_3_VSSe_3_ NCs as compared to the Cu_3_VS_2_Se_2_ NCs caused a shift in the Cu_3_VSSe_3_ peaks to higher 2θ angles than that expected from the Se composition. The crystallite sizes of the CVSSe NC compositions, as determined from the Scherrer formula are summarized in the Supplementary Materials (Figure S1). The Rietveld refinement for a representative CVSSe NC composition is shown in the Supplementary Materials (Figure S2) which demonstrates that the experimental XRD data closely matches with the peaks of pure cubic phase Cu_3_VS_4_. In addition, the peak positions for CuI, space group P4/nmm (129) at 2θ values of 28.51°, 32.84°, 36.98°, 41.01°, 47.57°, 51.13°, 56.00°, and 64.22° are shown on the plot. The peak positions for CuS, space group F$\bar{4}$3m (216) at 30.00°, 34.49°, 50.00°, 59.80°, and 62.10° are shown on the plot. Peak positions for the hexagonal P6_3_/mmc (194) phase of CuS are also shown in the plot. Notably, all of the CVSSe NC compositions exhibit XRD peaks distinct from the CuI and CuS reference peaks, such as the (010) peak for the cubic phase that is seen in the CVSSe NCs.

### 3.2. TEM and STEM Characterization

**Figure 2:**
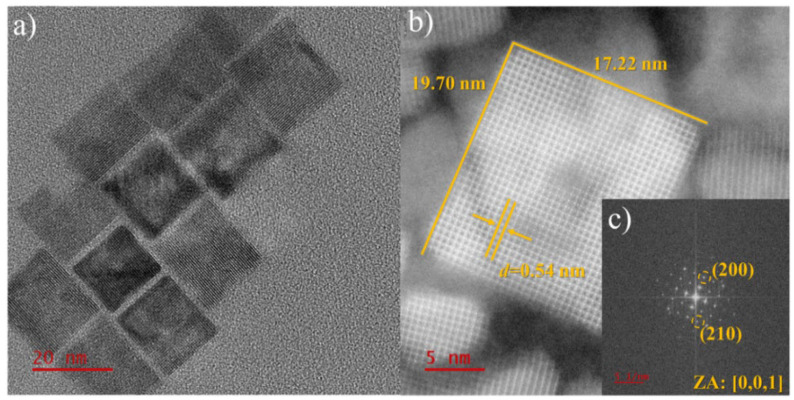
TEM and STEM characterization of Cu_3_VS_4_ NCs. (**a**) TEM, (**b**) STEM, and (**c**) corresponding FFT images of cubed-shaped Cu_3_VS_4_ NCs. Atomic resolution STEM image (**b**) shows the atomic arrangement of identified zone axis [0, 0, 1]. The zone axis is confirmed by indexing the lattice planes in the FFT diffraction patterns.

In conjunction with crystal phase analysis via XRD, the size, crystallinity, and morphology of the CVSSe NCs have been investigated via TEM and STEM. The end-member Cu_3_VS_4_ NCs are highly uniform in size and shape with a cubic morphology and an edge length of 19.5 ± 1.9 nm, based on the TEM images (Figure 2a,b and Figure S3). Figure 2b presents the high-resolution STEM image of a Cu_3_VS_4_ NC cube with a size of 19.7 nm × 17.2 nm. This is slightly smaller but consistent with the ensemble-averaged size of 25.3 ± 8.3 nm from XRD, given the different sampling scales of the two techniques. The clear lattice fringes in the STEM image indicate the high crystallinity of the NCs. The measured lattice fringe *d*-spacing of 0.536 ± 0.002 nm corresponds to the (010) plane of cubic Cu_3_VS_4_. Figure 2c (inset) shows the fast Fourier transform (FFT) and exhibits bright spots that correspond to (200) and (210) reflections of cubic Cu_3_VS_4_, confirming the crystalline nature of the NCs. Furthermore, the elemental composition analysis from Figures S4 and S5 and Table S1 confirms the expected atomic Cu:V:S composition ratio of 3:1:4.

**Figure 3:**
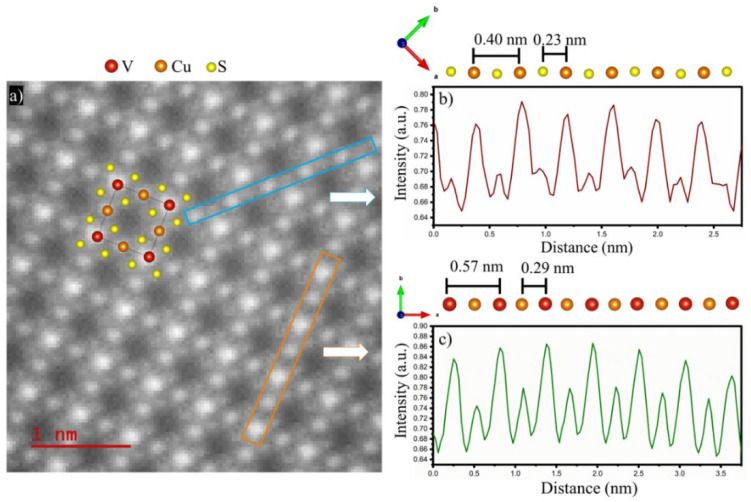
Cu_3_VS_4_ NCs. (**a**) Z-contrast image of Cu_3_VS_4_ in the [100] direction. The bright spots represent Cu, V, and S atoms in their respective atomic positions, (**b**) line profile of the Cu-S bonding column, (**c**) line profile of the Cu-V bonding column.

STEM Z-contrast imaging reveals the crystal’s unit cell periodicity along the [100] direction in Figure 3. Line profile analysis shows interatomic distances of V-V (0.565 nm), Cu-V (0.299 nm), 1 Cu-Cu (0.582 nm), 2 Cu-Cu (0.401 nm), and Cu-S (0.227 nm), which are in good agreement with the reported crystallographic data [32].

**Figure 4:**
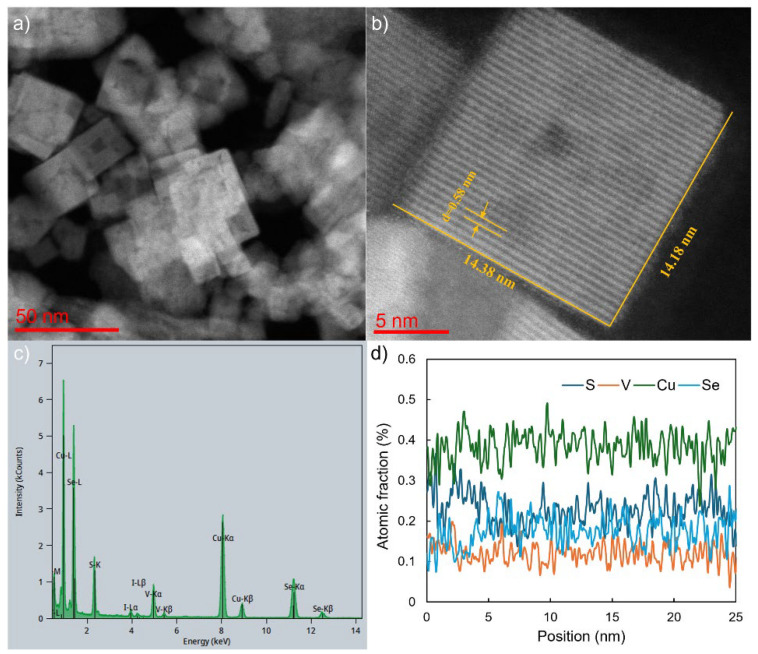
HAADF and EDX characterization of Cu_3_VS_2_Se_2_ NCs. (**a**) STEM image, (**b**) high-resolution STEM image, (**c**) EDX spectrum, and (**d**) EDX line profile.

Figure 4 shows STEM and high-angle annular dark field (HAADF) and EDX characterization of a representative CVSSe (Cu_3_VS_2_Se_2_) NC, which contains both S and Se. The NCs are also uniformly cubic in shape (Figure 4a) as observed in our TEM studies. They exhibit a size of ~14.18 nm × 14.38 nm. The NCs are also highly crystalline as indicated by the prominent lattice fringes (Figure 4b). The indicated lattice fringe *d*-spacing of 0.542 nm corresponds to the (010) plane of the cubic P$\bar{4}$3m phase. The elemental composition of the NCs was investigated via TEM-EDX (Figure 4c,d). The Cu, V, S, and Se signals are clearly visible in the EDX scan, as expected from the stoichiometry (Figure 4c). The presence of a small amount of iodide is likely due to remnant unreacted CuI that was not removed during the washing process. The chemical composition, Cu_3_VS_2_Se_1.7_, as detected from the EDX line spectra, closely matches the stoichiometry with a slight deficiency in Se (Figure 4d). Similar deficiencies in S, Se have been observed in PV devices containing Sb_2_(S, Se)_3_ thin films [36]. STEM, HAADF, and EDX characterization of the Cu_3_VS_3_Se and Cu_3_VSSe_3_ are shown in the Supplementary Materials (Figures S6 and S7).

### 3.3. Optical Properties of Cu_3_VS_x_Se_4−x_ NCs

**Figure 5:**
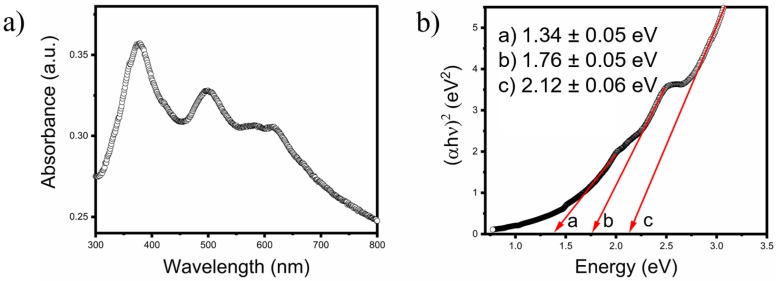
Experimental band gap of Cu_3_VS_4_ NCs. (**a**) UV-vis absorption spectrum of dispersed Cu_3_VS_4_ NCs. (**b**) The Tauc plot and extrapolation of the linear portion of the curve on the *x*-axis at zero absorption to determine the bandgap and transitions.

The optical properties of the CVSSe NCs have been investigated using UV-vis spectroscopy in the solution phase (Figure 5a). The end-member Cu_3_VS_4_ NCs exhibit three peaks in the visible range of the electromagnetic spectrum (375–650 nm), suggesting that the material is an IB semiconductor. The optical transitions observed have been extrapolated using the Tauc plot method by plotting (αhν)^2^ versus hν for direct bandgap, where α is the optical absorption coefficient, and *h* ν is the photon energy. The transition energy values have been determined by extrapolating the linear portion of the spectrum in the band edge region (Figure 5b). The extrapolated values for each transition are 1.34 ± 0.05 eV, 1.76 ± 0.05 eV, and 2.12 ± 0.06 eV, corresponding to the near-infrared (NIR-A) range, visible red region, and visible yellow regime of the electromagnetic spectrum, respectively. These band gaps are attributed to the electronic transitions from the VB to IB states within the NCs. The transition energy values for the indirect bandgaps are out of range, indicating that the material is a direct bandgap semiconductor. Notably, DFT-based calculations in the literature have predicted similar direct bandgaps and transition states for Cu_3_VS_4_ [18]. The UV-vis absorbance characterization and Tauc plot for IB band gap for other CVSSe NC compositions are shown in the Supplementary Materials (Figures S8–S11). Mantella et al. have performed DFT calculations to investigate the complex band structures of Cu_3_VS_4_ NCs in the pure cubic phase, and their results show that the theoretical band gap is in close agreement with the experimentally observed band gap obtained from the Tauc plots. Notably, the CVSSe NCs reported in this manuscript also exhibit a pure cubic phase and three intermediate band gaps that can be tuned by varying the Se content, but the overall optical property of these materials is similar to those of Cu_3_VS_4_ NCs. Consequently, the experimental Tauc plot is used to determine the band gap of the Cu_3_VS_4_ as well as the Cu_3_VS_x_Se_4−x_ NCs [18]. The IB characteristics of CVSSe NCs are further examined using electronic structure calculations based on DFT and HSE06. The results reveal an indirect band gap for all compositions. While no clearly isolated intermediate band is observed within the fundamental gap, the density of states exhibits a low-DOS region between the two high DOS peaks associated with the valence and conduction bands. When considered together with the multiple absorption features observed experimentally, this electronic structure is consistent with an IB-like interpretation for these materials. However, definite confirmation of IB characteristics in these materials will require additional measurement, such as photoluminescence excitation spectroscopy and photoconductivity-coupled external quantum efficiency spectroscopy, which will be pursued in our future work.

**Figure 6:**
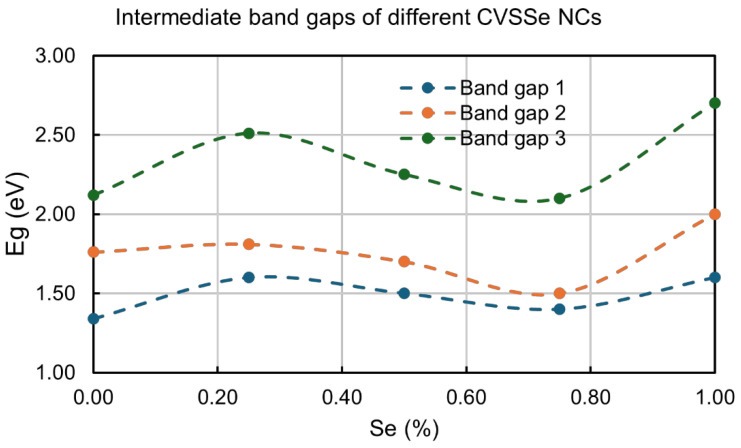
Plot showing the three intermediate direct band gaps of different composition Cu_3_VS_x_Se_4−x_ NCs.

Figure 6 and Table S2 summarize the intermediate band gaps of the different CVSSe NC compositions demonstrating that tunable band gap can be achieved across the visible and NIR regimes with these NCs. A slight decrease in band gap is observed with increasing Se concentration for the Cu_3_VS_3_Se, Cu_3_VS_2_Se_2_, and Cu_3_VSSe_3_ compositions, due to the larger size of the Se atoms [37]. However, this trend in optical band gap is not observed for the Cu_3_VS_4_ and Cu_3_VSe_4_ NCs, as several factors such as the nanocrystal size and defects of the NCs also influence their electronic properties. The corresponding UV-vis absorption plot and Tauc plots for the CVSSe NCs are shown in Figures S8–S11.

### 3.4. Theoretical Electronic Structure Studies

**Figure 7:**
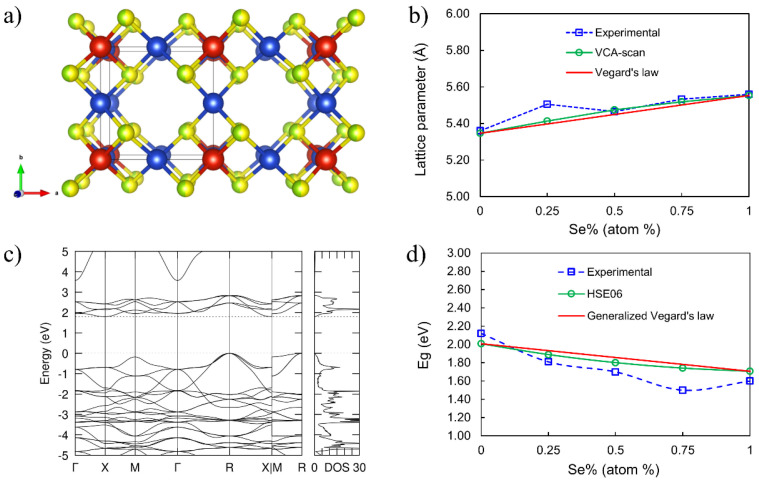
Structural and electronic properties of Cu_3_VS_x_Se_4−x_. (**a**) Perspective view of the 2 × 1 × 1 cubic structure of representative Cu_3_VS_2_Se_2_ showing equal occupancies for S (yellow) and Se (green) in the chalcogen sublattice. (**b**) Comparison of lattice parameters measured by XRD and calculated using SCAN meta-GGA functional, with the straight line representing Vegard’s law: a(x) = 5.346 + 0.207x. (**c**) Band structure and density of states for representative Cu_3_VS_2_Se_2_ showing an indirect band gap Eg of 1.80 eV, where the dashed lines indicate the valence band maximum (VBM, set at 0 eV) and conduction band minimum (CBM). (**d**) Comparison of band gaps measured by UV-vis spectroscopy and calculated using HSE06 hybrid functional, with the straight line representing the generalized Vegard’s law: Eg(x) = 2.007 − 0.301x.

To complement the experimental characterization, we carried out DFT calculations using the virtual crystal approximation to study the structural and electronic properties of the Cu_3_VS_x_Se_4−x_ (CVSSe) materials. Figure 7a depicts the relaxed atomic structure of the representative composition Cu_3_VS_2_Se_2_, showcasing the uniform distribution of S and Se atoms in the chalcogen sublattice. The relaxed atomic structures for the other four compositions are provided in Figures S12–S15 in the Supplementary Materials. The calculated lattice parameters, using the SCAN meta-GGA functional, exhibit a monotonic increase with increasing Se composition, which is in good agreement with experimental XRD measurements across the entire composition range (Figure 7b). This agreement confirms the validity of the structural models and provides strong support for the successful synthesis of CVSSe nanocrystals with controlled S/Se ratios. In addition, the calculated electronic structure of Cu_3_VS_2_Se_2_ (Figure 7c) reveals an indirect band gap of 1.80 eV, which closely matches the experimentally observed optical band gap obtained by UV-vis spectroscopy. Notably, the Fermi level lies only 0.168 eV above the valence band maximum (VBM) but 1.632 eV below the conduction band minimum (CBM), reflecting an asymmetric distribution of states near the band edges, an effect also observed for the four other compositions studied. This asymmetry arises from the larger band dispersion at the valence band edge relative to the conduction band edge, leading to a lower density of states near the VBM and resulting in an intrinsic p-type character for the series of sulvanite materials. See Figures S13–S15 in the Supplementary Materials for the band structures and density of states for the other four compositions. Figure 7d further shows that HSE06-calculated band gaps track well with experimental trends across the composition series. These results lend strong theoretical support to the experimentally observed structure–property relationships and confirm that the hot-injection synthesis method reliably produces phase-pure Cu_3_VS_x_Se_4−x_ NCs with tunable electronic properties.

The slight systematic offset between the experimentally observed band gaps and those calculated using Vegard’s law and HSE06 is expected. HSE06 calculations are primarily used to capture the compositional trend in the band gap across the CVSSe series, whereas a quantitative comparison with nanocrystal optical gaps would require explicit consideration of excitonic effects, surface states, and many-body interactions, for example, within GW or GW-BSE frameworks. These effects, together with nanocrystal-specific features not captured in bulk calculations, can contribute to the observed deviations. While the band gap generally shows a decreasing trend with increased Se substitution as expected because of the larger size of the Se atom, the Cu_3_VSe_4_ exhibits a higher band gap than expected [38]. This is likely due to the larger size of the Cu_3_VSe_4_ NCs as compared to the other CVSSe NC compositions (Figure S1). The CVSSe NCs synthesized using Cu(acac)_2_ precursor were less crystalline as compared to the ones synthesized with CuI and the S:Se composition deviated from the stoichiometry (Figure S16).

### 3.5. Photoluminescence Characterization of Cu_3_VS_x_Se_4−x_ NCs

**Figure 8:**
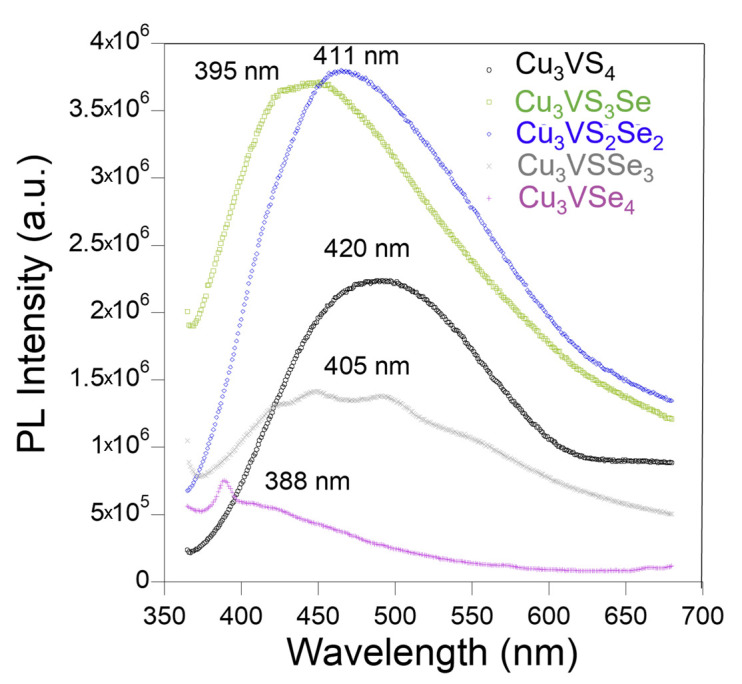
PL spectra of different Cu_3_VS_x_Se_4−x_ NCs. Excitation wavelength was 350 nm for the PL spectra.

The novel CVSSe IB semiconductor NCs also exhibit emission in the visible range (Figure 8). To investigate the composition-dependent photoluminescence (PL) emission, each of the CVSSe NC samples was excited at 350 nm. The PL peaks are attributed to the mid-gap charge trap induced by Cu vacancies near the VBM [39]. A slight blue shift is observed in the PL peaks of CVSSe NCs with increasing Se content in the NCs due to the smaller size of the NCs with Se substitution (Figure S1) [40]. This trend may be attributed to a combination of factors, including ligand packing on the NC surfaces, size effects, and defects in the crystal lattice of the CVSSe NCs (Figure S1). In general, the CVSSe NCs exhibit emission in the visible range and can be used for various PV and light-emission applications in the visible range. The Stokes shift, calculated from the difference in absorption and emission spectra, for Cu_3_VS_4_, Cu_3_VS_3_Se, Cu_3_VS_2_Se_2_, Cu_3_VSSe_3_, and Cu_3_VSe_4_ NCs are 0.411 eV, 0.344 eV, 0.163 eV, 0.290 eV, and 0.347 eV, respectively. The larger Stokes shift for the compositions with higher S content will be attractive for sensing and bio-imaging applications.

### 3.6. Photoresponse Properties of Cu_3_VS_4_ NCs

**Figure 9:**
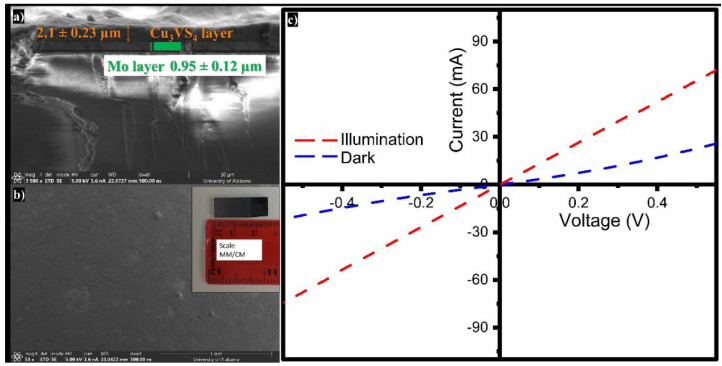
SEM images of (**a**) cross-sectional view of ~2.1 µm thick Cu_3_VS_4_ thin film fabricated on a Mo-coated glass substrate surface, (**b**) top-down view of the surface of the film with inset showing the fabricated device, and (**c**) current-voltage (I–V) characteristics of Cu_3_VS_4_ thin film structure under white light illumination at 100 mW cm^−2^ at dark and illuminated conditions.

The photoresponse of a Cu_3_VS_4_ thin film has been investigated in the dark and under illumination. The Cu_3_VS_4_ NCs are incorporated as an absorber layer in a photovoltaic heterostructure composed of soda-lime glass/Mo/Cu_3_VS_4_/Ag. The NCs (~150 mg) are resuspended in 30 mL of hexane to form a nano-ink solution. The nano-ink solution is sprayed and coated on the Mo substrate. The coated substrate is subsequently annealed in nitrogen atmosphere at 400 °C for 2 h. SEM analysis reveals that the fabricated thin film exhibits a continuous surface with thickness of 2.1 ± 0.2 µm (Figure 9).

To probe for photoconductivity, the fabricated thin film is connected to a potentiostat between the top (silver) and bottom (molybdenum) electrodes. The thin film is illuminated using a full spectrum Xenon lamp (50 W), and voltage input is increased from −0.5 to 0.5 V in steps. The generated current is measured in the dark and under illumination. The observed current-voltage (I-V) curve of the thin film reveals the Ohmic nature of the contact. Cu_3_VS_4_ exhibits a higher response under illumination as compared to the dark measurement. The fabricated thin film has an effective area of 1.58 cm^2^ and shows a net photocurrent of 46.55 mA. The responsivity, which is the photocurrent generated per unit power of the incident light used, is calculated to be 0.26 AW^−1^. This high responsivity of the Cu_3_VS_4_ thin films is comparable to that of crystalline silicon photodetectors and other semiconductors [41,42,43,44,45,46]. Furthermore, the fabricated thin film has a current conversion efficiency of 14.7% with respect to dark current, where the current conversion efficiency is incident photon-to-current efficiency.

The photocurrent results are summarized in Table 1. These results suggest that thin films made of Cu_3_VS_4_ NCs and potentially CVSSe NCs are suitable for producing higher photocurrents in solar energy conversion devices. In summary, promising results from a photoconductive test structure are presented in this study for the Cu_3_VS_4_ NC-based thin films. Recently, Syu et al. reported the fabrication of co-sputtered Cu_3_VS_4_ thin film solar cells, achieving a maximum power conversion efficiency of 3.11% [47]. Building on this work, further improvements are anticipated for the CVSSe NC films, which are additionally expected to provide tunable photocurrent. Our future endeavors will involve integrating the CVSSe NCs as absorber layers in a full p-n junction solar cell unit for characterizing the open-circuit voltage (V_OC_), current density, and power conversion efficiency. Recently, Nushin et al., predicted a V_OC_ of 0.94 V, short circuit current of 34.39 mA/cm^2^, fill factor of 87.47%, and 28.33% power conversion efficiency through computational evaluation using Cu_3_VS_4_ as the absorber layer, a ZnS transparent layer, and V_2_O_5_ as the back surface field layer [48]. Building on this work, our ongoing studies are aimed at further characterizing the CVSSe IB materials in solar cell diagnostic devices through experiments.

## 4. Conclusions

In summary, we successfully synthesized a new class of highly crystalline colloidal Cu_3_VS_x_Se_4−x_ NCs with a pure cubic phase and cubic morphology, exhibiting composition-dependent tunable intermediate band gaps and photoluminescence (PL) emission. The Cu_3_VS_4_ NCs exhibit an average size of approximately19.5 nm, while the average sizes of the CVSSe NCs across different compositions range from 10.9 nm to 19.1 nm. The structure, phase, morphology, and purity of the synthesized NCs were thoroughly characterized using X-ray diffraction (XRD), scanning electron microscopy (SEM), high-resolution transmission electron microscopy (HRTEM), and scanning transmission electron microscopy (STEM) techniques. Atomic resolution STEM imaging confirmed the highly crystalline nature of the NCs, and Z-contrast imaging enabled the estimation of interatomic distances within the unit cell. Ultraviolet-visible (UV-vis) absorption analysis of Cu_3_VS_4_ revealed three distinct bands with allowed transition values of 1.34 eV, 1.76 eV, and 2.10 eV. The various CVSSe NC compositions also exhibited three intermediate band gaps spanning the visible and near-infrared (NIR) regimes, with a slight decrease in band gap observed with increased Se substitution, attributed to the larger size of the Se atom. First-principles DFT calculations based on virtual crystal approximation gave good agreement in lattice parameters and band gaps across the CVSSe series, lending confidence that the targeted phases and compositions have been successfully realized. The CVSSe NCs displayed PL emission in the visible range with a 350 nm excitation source. Furthermore, a Cu_3_VS_4_ thin film was fabricated and tested for photoconductivity, demonstrating good photoresponse and responsivity characteristics in the current-voltage (I–V) measurement. Notably, a photon-to-current conversion efficiency of 14.7% was achieved with this material. These results pave the way for the development of next-generation photovoltaic devices incorporating intermediate band gap semiconductors. In our future studies, layered structures of these novel IB materials will be explored to further enhance the current conversion efficiency.

## Figures and Tables

**Table 1:** Photocurrent and responsivity of Cu_3_VS_4_ thin film electrode.

Thin Film (Material)	Bias Voltage (V)	Photocurrent (mA)	Responsivity (A W^−1^)	Photosensitivity	Efficiency (%)
Cu_3_VS_4_	0.5	46.55	0.26	1.83	14.7

## Data Availability

The data supporting this article have been included as part of the Supplementary Information. This manuscript has been authored by UT-Battelle, LLC, under Contract No. DE-AC0500OR22725 with the U.S. Department of Energy. The United States Government retains and the publisher, by accepting the article for publication, acknowledges that the United States Government retains a non-exclusive, paid-up, irrevocable, world-wide license to publish or reproduce the published form of this manuscript, or allow others to do so, for the United States Government purposes. The Department of Energy will provide public access to these results of federally sponsored research in accordance with the DOE Public Access Plan (http://energy.gov/downloads/doe-public-access-plan, accessed on 4 January 2026).

## Supplementary Materials

The following supporting information can be downloaded at: https://www.mdpi.com/article/10.3390/nano16020082/s1, Scheme S1. A schematic representation of the hot-injection synthesis scheme to obtain Cu_3_VS_x_Se_4−x_ NCs. Figure S1. Trend in sizes of Cu_3_VS_x_Se_4−x_ NCs w.r.t. Se composition in the NCs, obtained from XRD measurements. Figure S2. Rietveld refinement of Cu_3_VS_2_Se_2_ NCs showing close agreement with the cubic P$\bar{4}$3m phase. Figure S3. Statistical size analysis of Cu_3_VS_4_ NCs with corresponding TEM image. Figure S4. Energy dispersive X-ray (EDX) mapping analysis of Cu_3_VS_4_ NCs. Figure S5. EDX spectrum of Cu_3_VS_4_ NCs. Table S1. Elemental composition of Cu_3_VS_4_ NCs determined by EDX. Figure S6. HAADF and EDX characterization of Cu_3_VS_3_Se NCs. (a) STEM image, (b) high-resolution STEM image, (c) EDX spectrum, and (d) EDX line profile. Figure S7. HAADF and EDX characterization of Cu_3_VSSe_3_ NCs. (a) STEM image, (b) high-resolution STEM image, (c) EDX spectrum, and (d) EDX line profile. Figure S8. Experimental band gap of Cu_3_VS_3_Se NCs. (a) Ultraviolet visible (UV-vis) absorbance plot and (b) Tauc plot showing direct intermediate (IB) band gaps. Figure S9. Experimental band gap of Cu_3_VS_2_Se_2_ NCs. (a) UV-vis absorbance plot and (b) Tauc plot showing direct IB band gaps. Figure S10. Experimental band gap of Cu_3_VSSe_3_ NCs. (a) UV-vis absorbance plot and (b) Tauc plot showing direct IB band gaps. Figure S11. Experimental band gap of Cu_3_VSe_4_ NCs. (a) UV-vis absorbance plot and (b) Tauc plot showing direct IB band gaps. Table S2. Table showing the intermediate band gaps of the different CVSSe NC compositions. Figure S12. Band gap and density of states (DOS) of Cu_3_VS_4_ NCs. Figure S13. Electronic structure of Cu_3_VS_3_Se NCs. (a) Structure and (b) band gap and DOS. Figure S14. Electronic structure of Cu_3_VSSe_3_ NCs. (a) Structure and (b) band gap and DOS. Figure S15. Electronic structure of Cu_3_VSe_4_ NCs. (a) Structure and (b) band gap and DOS. Figure S16. XRD characterization of different Cu_3_VS_x_Se_4−x_ NCs synthesized using Cu(acac)_2_ precursor.
