# Supplementary material for: Intermediate Bandgap (IB) Cu3VSxSe4−x Nanocrystals as a New Class of Light Absorbing Semiconductors
Source: Nanomaterials (Basel). 2026 Jan 7;16(2):82. doi: 10.3390/nano16020082 (PMC12844221; doi:10.3390/nano16020082)
Supplement: Supplementary file 1 [file nanomaterials-16-00082-s001.zip › nanomaterials-4052551-supplementary.pdf]

# Supporting Information

## Intermediate bandgap (IB) $Cu_3VS_xSe_{4-x}$ nanocrystals as a new class of light absorbing semiconductors

Jose J. Sanchez Rodriguez [1], Soubantika Palchoudhury [2,*], Jingsong Huang [3], Daniel Speed [1], Elizaveta Tiukalova [3], Godwin Mante [2], Jordan Hachtel [3] and Arunava Gupta [1,4,*]

*1.Department of Chemistry and Biochemistry, The University of Alabama, Tuscaloosa, AL 35401, USA*

*2. Chemical and Materials Engineering, University of Dayton, Dayton, OH 45469, USA*

*3. Center for Nanophase Materials Sciences, Oak Ridge National Laboratory, Oak Ridge, TN 37830, USA*

*4. Department of Chemical and Nano Engineering, University of California San Diego, La Jolla, CA 92093, USA*

** Correspondence: spalchoudhury1@udayton.edu (S.P.); arungupta@ucsd.edu (A.G.); Tel.: +1-937-229-3194 (S.P.); +1-2053294402 (A.G.)*

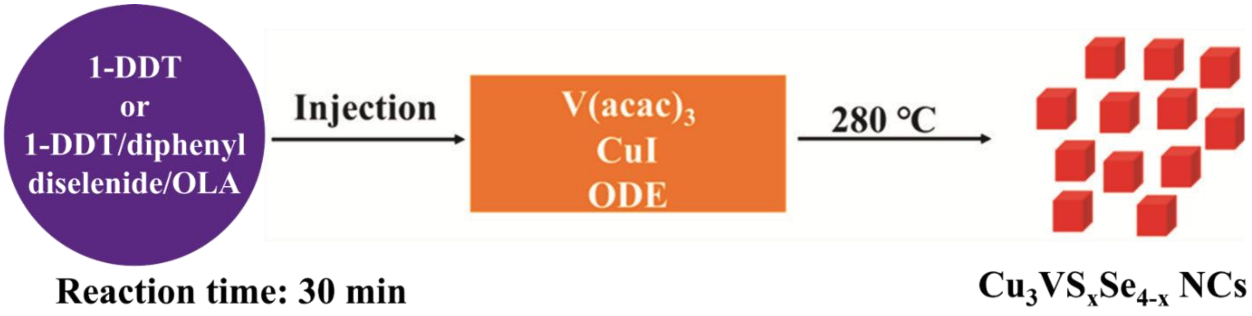


**Scheme S1.** A schematic representation of the hot-injection synthesis scheme to obtain $Cu_3VS_xSe_{4-x}$ NCs.

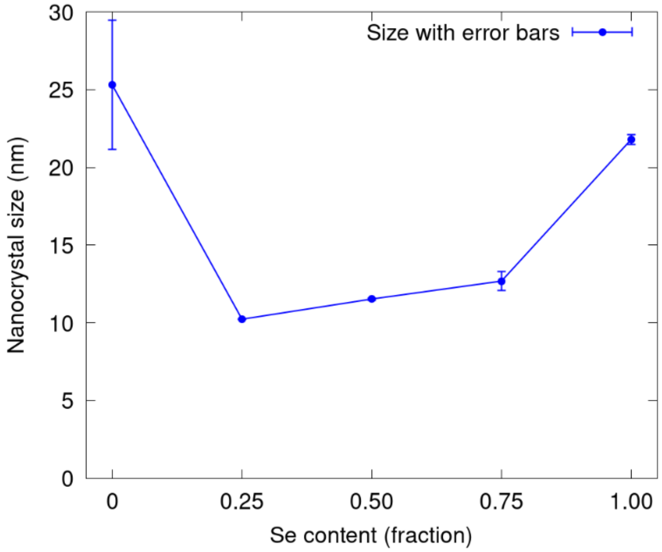


**Figure S1.** Trend in sizes of $Cu_3VS_xSe_{4-x}$ NCs w.r.t. Se composition in the NCs, obtained from XRD measurements.

Fig. S2 presents the Rietveld fit of $Cu_2VS_2Se_2$ NCs obtained using GSAS-II software. The refinement was performed over a 2θ range of 10 – 90 degrees. As a reference, $Cu_3VS_4$ with a pure $P\bar{4}3m$ phase was used for comparison, with the corresponding cif file obtained from the Materials Project. The fit was conducted using size, microstrain, and atoms as primary variables, along with background correction. The refinement reveals a close match of the experimental peaks with reference. The difference in peak intensities of the peaks between the observed or experimental

XRD and the theoretical XRD spectra can be attributed to several factors, including the preferred orientation factor and absorption multiplier employed in the Rietveld refinement. The errors in the Rietveld refinement are represented by the bottom curve, which shows the intensity difference between the observed and calculated XRD peaks normalized by the standard deviation.

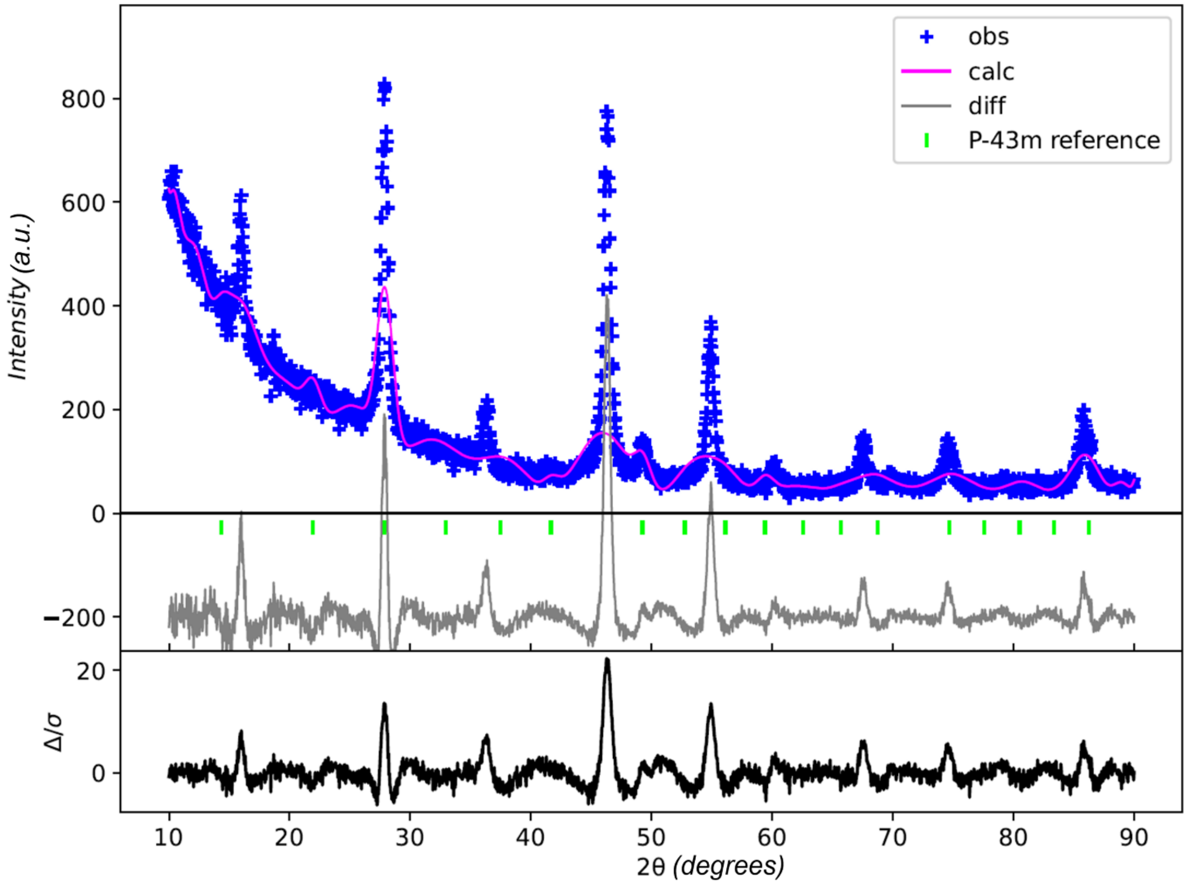


**Figure S2.** Rietveld refinement of $Cu_3VS_2Se_2$ NCs showing close agreement with the cubic P4̄3m phase. The top panel shows experimental and calculated XRD plot, while the bottom panel shows the difference between the observed XRD peaks and those calculated via Rietveld refinement normalized by the standard uncertainty.

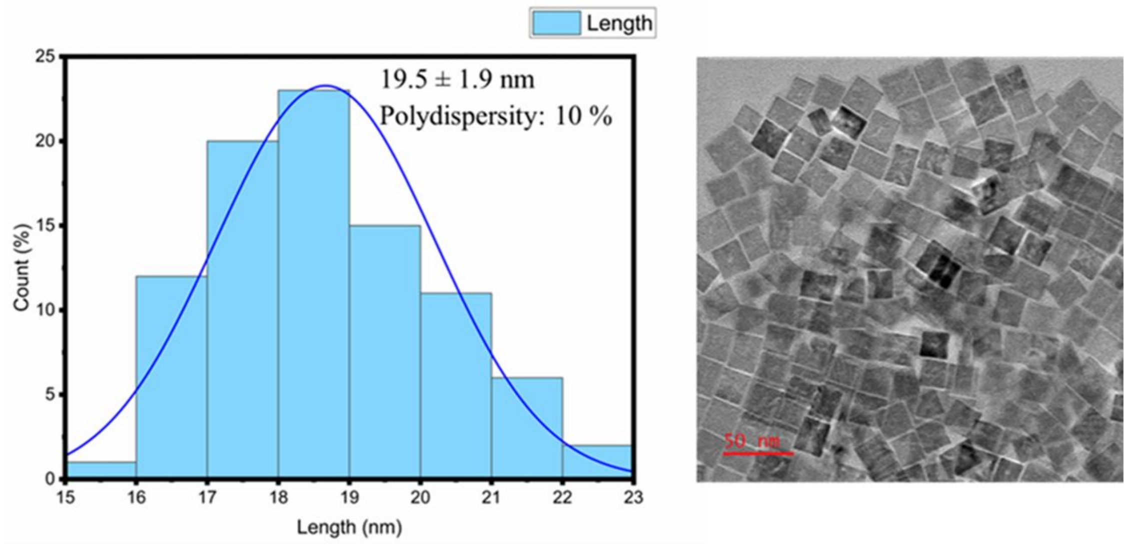


**Figure S3.** Statistical size analysis of $Cu_3VS_4$ NCs with corresponding TEM image.

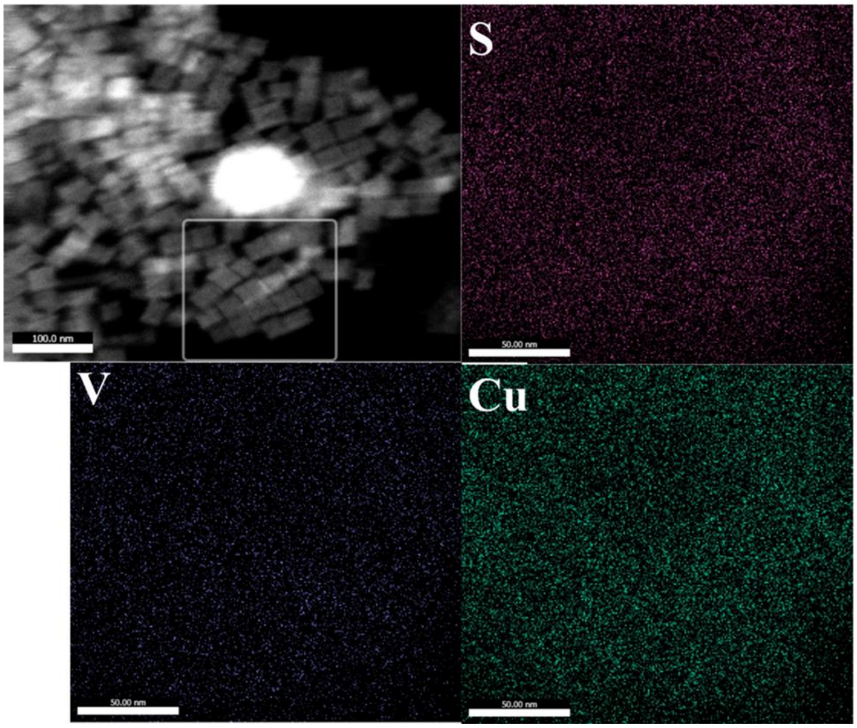


**Figure S4.** Energy dispersive X-ray (EDX) mapping analysis of $Cu_3VS_4$ NCs.

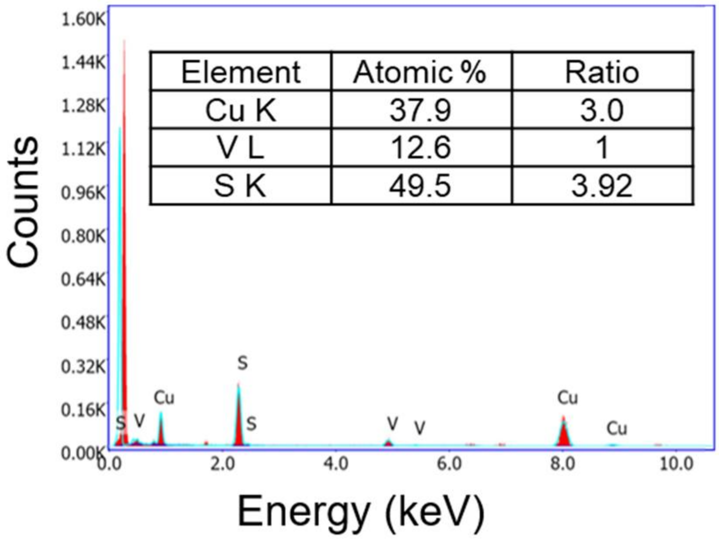


**Figure S5.** EDX spectrum of $Cu_3VS_4$ NCs.

Table S1. Elemental composition of $Cu_3VS_4$ NCs determined by EDX.

| Element | Atomic % | Ratio |
|---|---|---|
| Cu K | 37.9 | 3.0 |
| V L | 12.6 | 1 |
| S K | 49.5 | 3.92 |

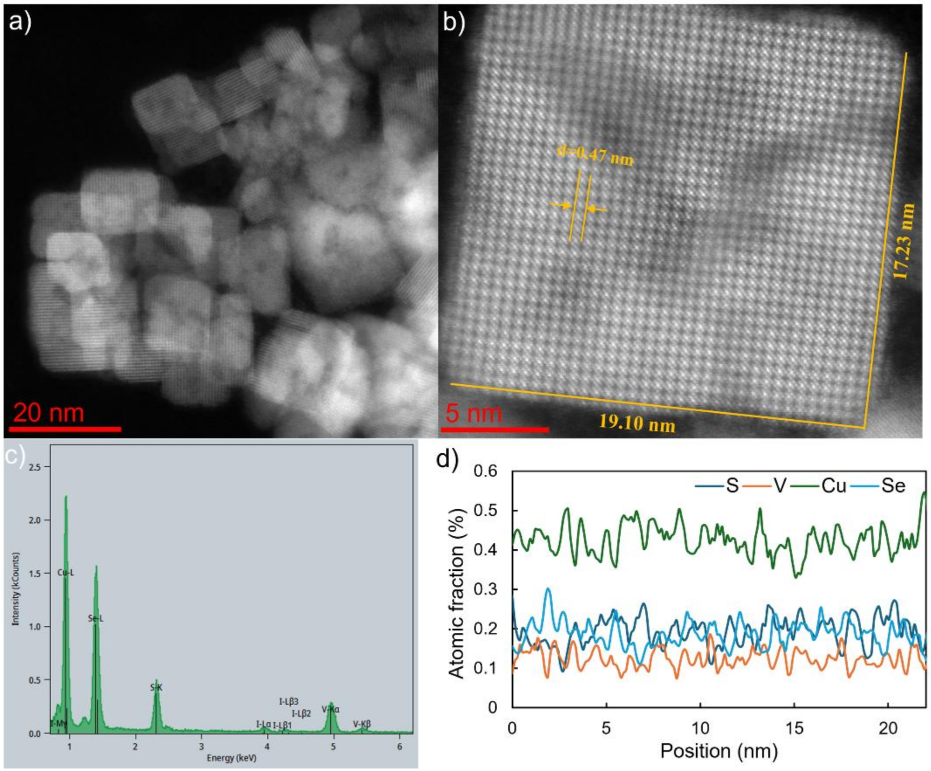


**Figure S6.** HAADF and EDX characterization of $Cu_3VS_3Se$ NCs. (a) STEM image, (b) high-resolution STEM image, (c) EDX spectrum, and (d) EDX line profile.

The $Cu_3VS_3Se$ NCs are also cubic in shape and highly crystalline (Fig. S6a). Prominent lattice fringes are visible in the high-resolution STEM image (Fig. S6b). The NCs are ~ 19.10 nm x 17.23 nm in size with a lattice spacing of 0.47 nm corresponding to the (010) plane of cubic $P\overline{4}3m$ phase. The elemental composition of the NCs was investigated via TEM-EDX and shows prominent peaks of Cu, V, S, and Se, indicating the presence of these elements in the NC composition (Fig. S6c). The small peak from I is due to remnant CuI that was not removed during the cleaning process. The atomic line profile from the EDX shows a composition ($Cu_3VS_{2.4}Se_{1.5}$) close to stoichiometry for these NCs (Fig. S6d).

Fig. S7 shows the HAADF-STEM image and EDX characterization of $Cu_3VSSe_3$ NCs. The NCs are cubic in shape with a size of ~ 10.90 nm x 12.41 nm (Figs. S7a-b). The

lattice spacing of 0.46 nm corresponds to the (010) plane of cubic P$\overline{4}$3m phase (Fig. S7b). The presence of all four expected elements, Cu, V, S, and Se are detected in the EDX spectra of these NCs (Fig. S7c). The EDX atomic % line profile, that shows the elemental composition across the single NC imaged in the high-resolution STEM, matches closely with the stoichiometric composition (Fig. S7d) with a composition of $Cu_3VS_{1.2}Se_{2.6}$.

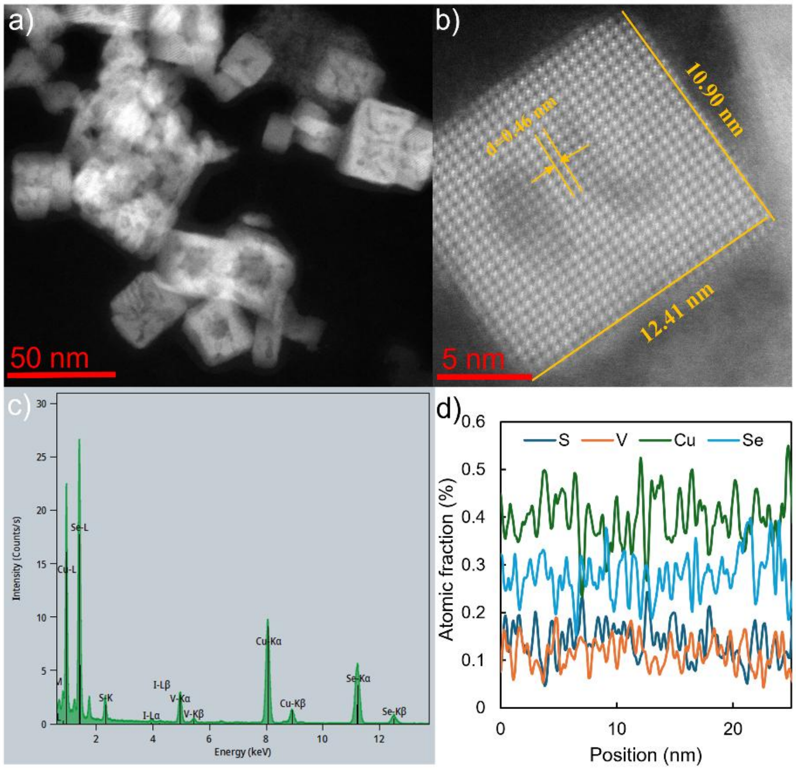


**Figure S7.** HAADF and EDX characterization of $Cu_3VSSe_3$ NCs. (a) STEM image, (b) high-resolution STEM image, (c) EDX spectrum, and (d) EDX line profile.

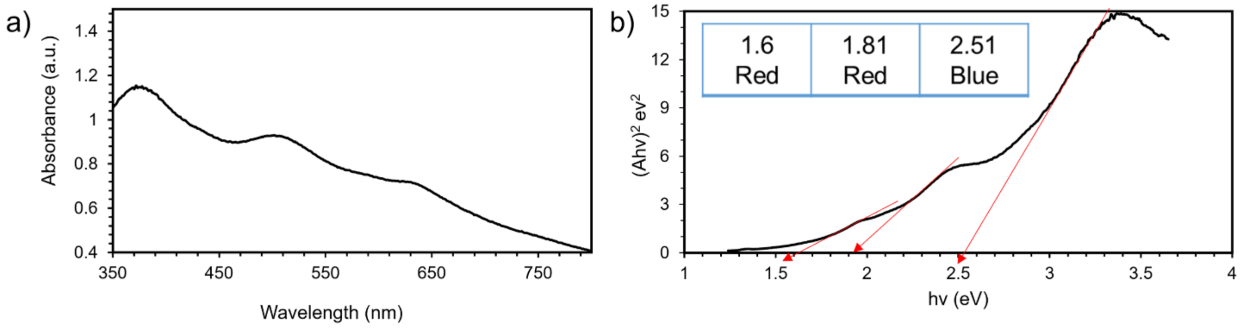

**Figure S8.** Experimental band gap of $Cu_3VS_3Se$ NCs. (a) Ultraviolet visible (UV-vis) absorbance plot and (b) Tauc plot showing direct intermediate (IB) band gaps.

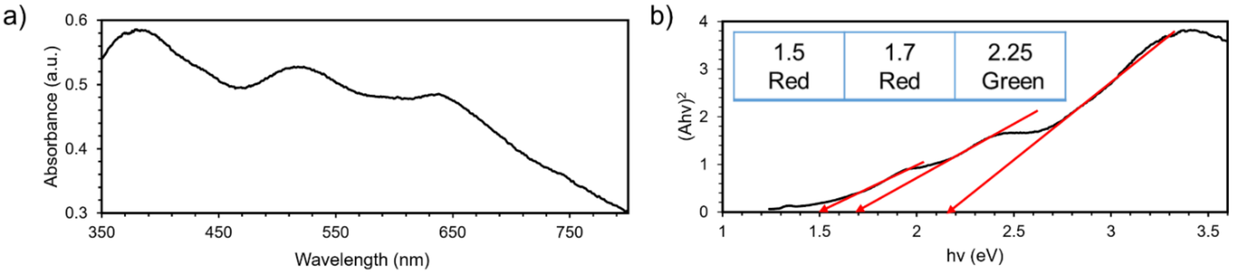


**Figure S9.** Experimental band gap of $Cu_3VS_2Se_2$ NCs. (a) UV-vis absorbance plot and (b) Tauc plot showing direct IB band gaps.

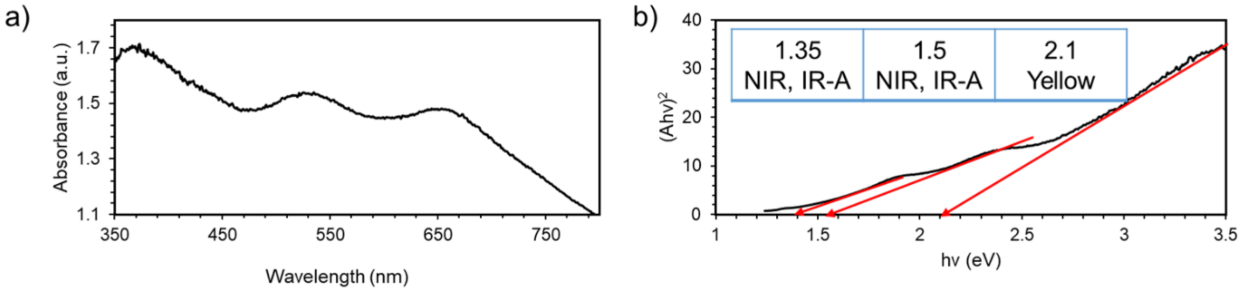


**Figure S10.** Experimental band gap of $Cu_3VSSe_3$ NCs. (a) UV-vis absorbance plot and (b) Tauc plot showing direct IB band gaps.

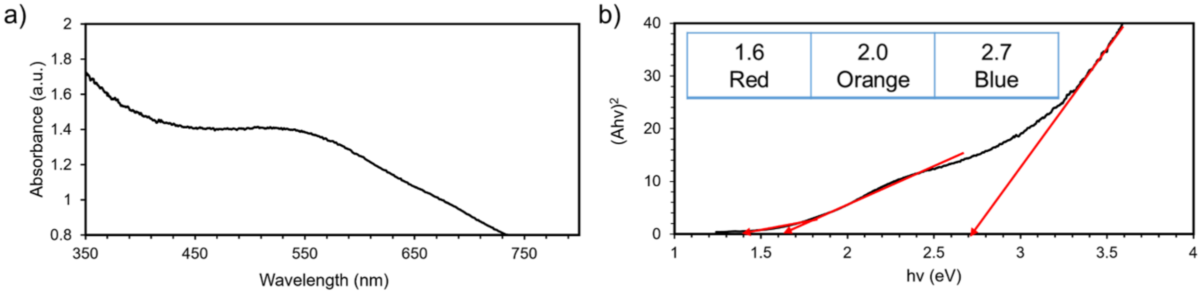


**Figure S11.** Experimental band gap of $Cu_3VSe_4$ NCs. (a) UV-vis absorbance plot and (b) Tauc plot showing direct IB band gaps.

Table S2. Table showing the intermediate band gaps of the different CVSSe NC compositions.

| **$Cu_3VS_xSe_{4-x}$ IB NC composition** | **Direct band gap 1 (eV)** | **Direct band gap 2 (eV)** | **Direct band gap 3 (eV)** |
|---|---|---|---|
| $Cu_3VS_4$ | 1.34<br>NIR-A | 1.76<br>Red | 2.12<br>Yellow |
| $Cu_3VS_3Se$ | 1.60<br>Red | 1.81<br>Red | 2.51<br>Blue |
| $Cu_3VS_2Se_2$ | 1.50<br>NIR-A | 1.70<br>Red | 2.25<br>Green |
| $Cu_3VSSe_3$ | 1.40<br>NIR-A | 1.50<br>NIR-A | 2.10<br>Yellow |
| $Cu_3VSe_4$ | 1.60<br>Red | 2.00<br>Orange | 2.70<br>Blue |

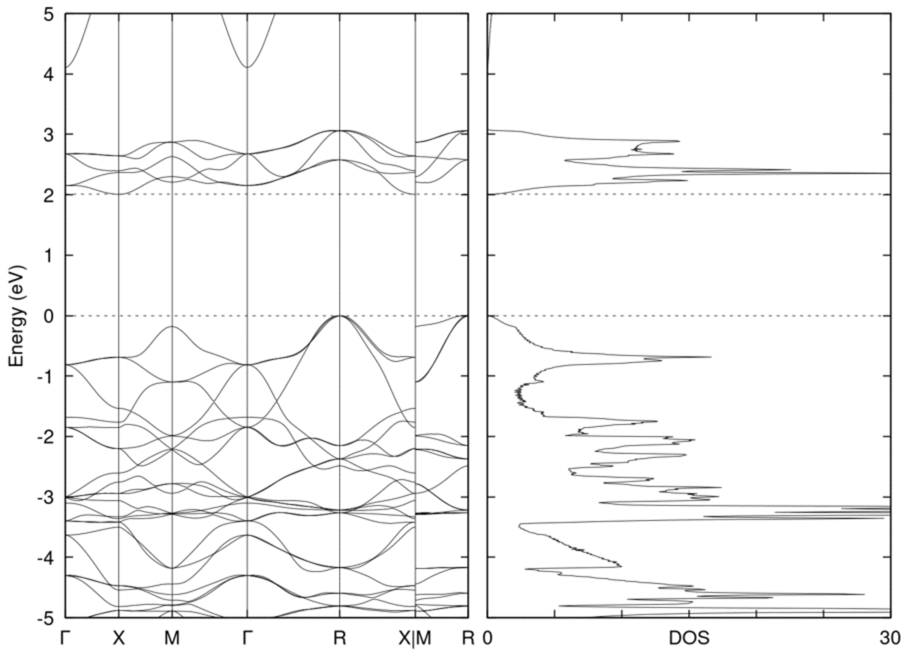


**Figure S12.** Band gap and density of states (DOS) of $Cu_3VS_4$ NCs.

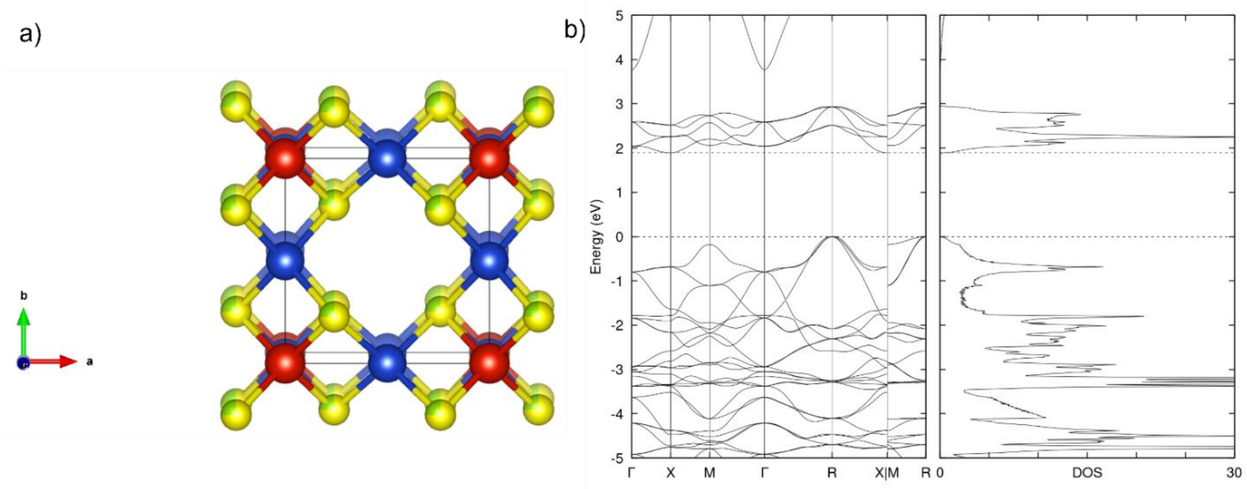


**Figure S13.** Electronic structure of $Cu_3VS_3Se$ NCs. (a) Structure and (b) band gap and DOS.

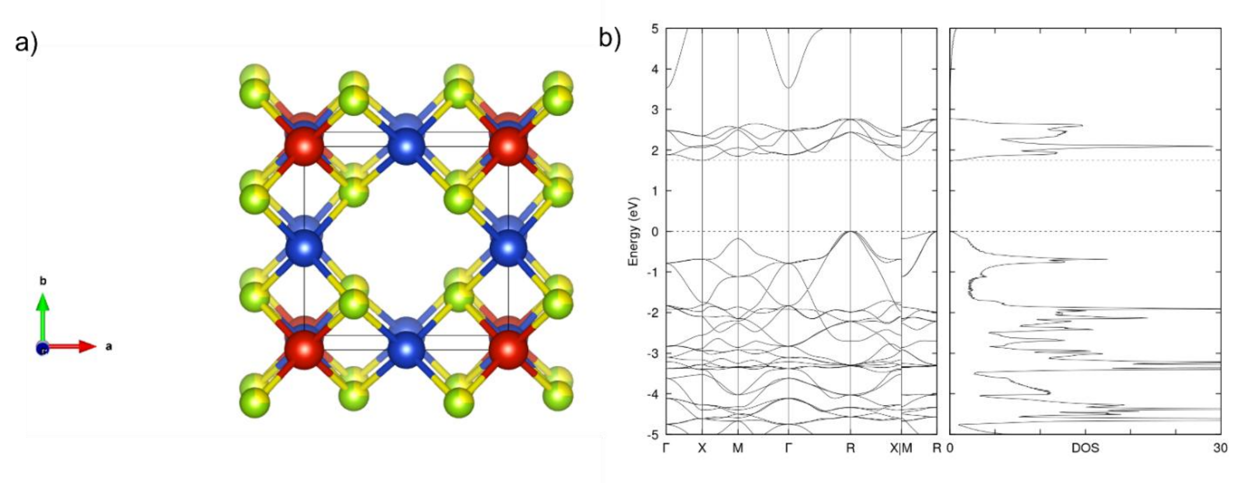


**Figure S14.** Electronic structure of $Cu_3VSSe_3$ NCs. (a) Structure and (b) band gap and DOS.

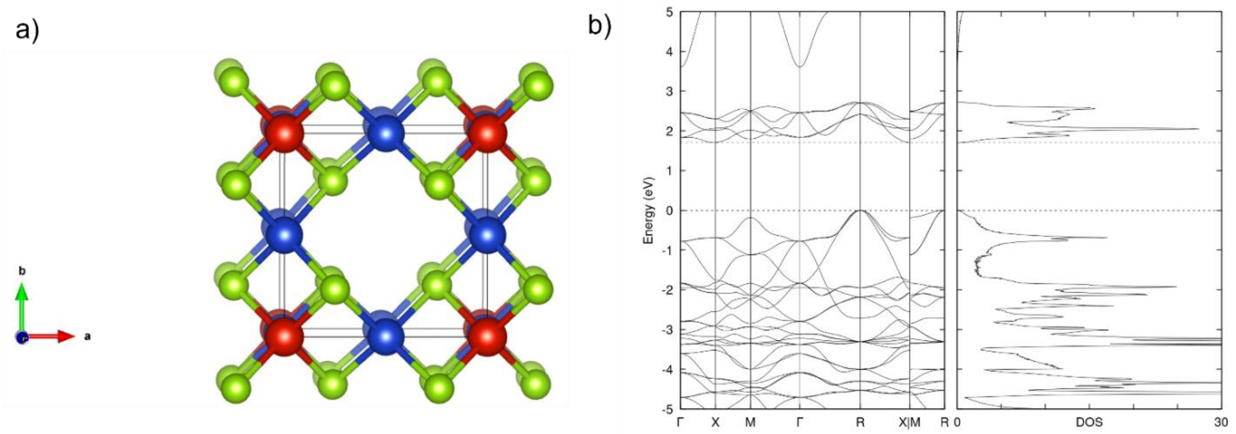


**Figure S15.** Electronic structure of $Cu_3VSe_4$ NCs. (a) Structure and (b) band gap and DOS.

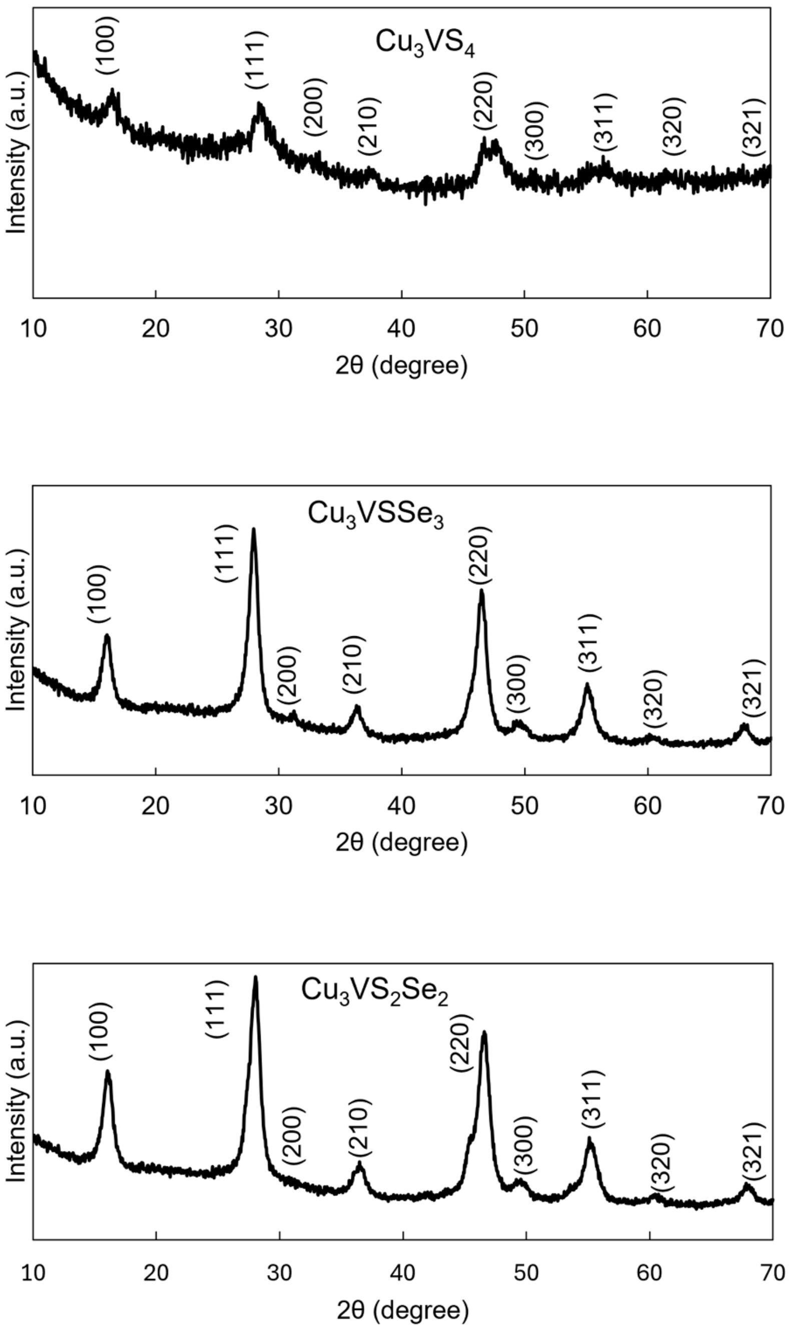


**Figure S16.** XRD characterization of different $Cu_3VS_xSe_{4-x}$ NCs synthesized using $Cu(acac)_2$ precursor.
